# Lethal Injection for Execution: Chemical Asphyxiation?

**DOI:** 10.1371/journal.pmed.0040156

**Published:** 2007-04-24

**Authors:** Teresa A Zimmers, Jonathan Sheldon, David A Lubarsky, Francisco López-Muñoz, Linda Waterman, Richard Weisman, Leonidas G Koniaris

**Affiliations:** 1 Dewitt Daughtry Family Department of Surgery, University of Miami Miller School of Medicine, Miami, Florida, United States of America; 2 Department of Cell Biology and Anatomy, University of Miami Miller School of Medicine, Miami, Florida, United States of America; 3 Devine, Connell & Sheldon, P.L.C., Fairfax, Virginia, United States of America; 4 Department of Anesthesiology, Perioperative Medicine, and Pain Management, University of Miami Miller School of Medicine, Miami, Florida, United States of America; 5 Department of Management, University of Miami School of Business, Miami, Florida, United States of America; 6 Department of Pharmacology, University of Alcalá, Madrid, Spain; 7 Department of Comparative Medicine and Pathology, University of Miami Miller School of Medicine, Miami, Florida, United States of America; 8 Florida Poison Center–Miami, University of Miami Miller School of Medicine, Miami, Florida, United States of America; Harvard Medical School, United States of America

## Abstract

**Background:**

Lethal injection for execution was conceived as a comparatively humane alternative to electrocution or cyanide gas. The current protocols are based on one improvised by a medical examiner and an anesthesiologist in Oklahoma and are practiced on an ad hoc basis at the discretion of prison personnel. Each drug used, the ultrashort-acting barbiturate thiopental, the neuromuscular blocker pancuronium bromide, and the electrolyte potassium chloride, was expected to be lethal alone, while the combination was intended to produce anesthesia then death due to respiratory and cardiac arrest. We sought to determine whether the current drug regimen results in death in the manner intended.

**Methods and Findings:**

We analyzed data from two US states that release information on executions, North Carolina and California, as well as the published clinical, laboratory, and veterinary animal experience. Execution outcomes from North Carolina and California together with interspecies dosage scaling of thiopental effects suggest that in the current practice of lethal injection, thiopental might not be fatal and might be insufficient to induce surgical anesthesia for the duration of the execution. Furthermore, evidence from North Carolina, California, and Virginia indicates that potassium chloride in lethal injection does not reliably induce cardiac arrest.

**Conclusions:**

We were able to analyze only a limited number of executions. However, our findings suggest that current lethal injection protocols may not reliably effect death through the mechanisms intended, indicating a failure of design and implementation. If thiopental and potassium chloride fail to cause anesthesia and cardiac arrest, potentially aware inmates could die through pancuronium-induced asphyxiation. Thus the conventional view of lethal injection leading to an invariably peaceful and painless death is questionable.

## Abstract 2

Data from executions in the US suggest that current lethal injection protocols do not effect death through the mechanisms intended, and that potentially aware inmates could die through pancuronium-induced asphyxiation.

## Editors' Summary

**Background.:**

Lethal injection is a common form of execution in a number of countries, most prominently the US and China. The protocols currently used in the US contain three drugs: an ultrashort-acting barbiturate, thiopental (which acts as an anesthetic, but does not have any analgesic effect); a neuromuscular blocker, pancuronium bromide (which causes muscle paralysis); and an electrolyte, potassium chloride (which stops the heart from beating). Each of these drugs on its own was apparently intended by those who derived the protocols to be sufficient to cause death; the combination was intended to produce anesthesia then death due to respiratory and cardiac arrest. Following a number of executions in the US, however, it has recently become apparent that the regimen as currently administered does not work as efficiently as intended. Some prisoners take many minutes to die, and others become very distressed.

**Why Was This Study Done?:**

It is possible that one cause of these difficulties with the injections is that the staff administering the drugs are not sufficiently competent; doctors and nurses in the US are banned by their professional organizations from participating in executions and hence most personnel have little medical knowledge or skill. Alternatively, the drug regimens used might not be effective; it is not clear whether they were derived in any rational way. The researchers here wanted to investigate the scientific basis for the protocols used.

**What Did the Researchers Do and Find?:**

They analyzed data from some of the few states (North Carolina and California) that release information on executions. They also assessed the regimens with respect to published data from clinical, laboratory, and veterinary animal studies. The authors concluded that in the current regimen thiopental might not be fatal and might be insufficient to induce surgical anesthesia for the duration of the execution, and that potassium chloride does not reliably induce cardiac arrest. They conclude therefore that potentially aware inmates could die through asphyxiation induced by the muscle paralysis caused by pancuronium.

**What Do These Findings Mean?:**

The authors conclude that even if lethal injection is administered without technical error, those executed may experience suffocation, and therefore that “the conventional view of lethal injection as an invariably peaceful and painless death is questionable.” The Eighth Amendment of the US Constitution prohibits cruel and unusual punishment. The results of this paper suggest that current protocols used for lethal injection in the US probably violate this amendment.

**Additional Information.:**

Please access these Web sites via the online version of this summary at http://dx.doi.org/10.1371/journal.pmed.0040156.

- In a linked editorial the *PLoS Medicine* editors discuss this paper further and call for the abolition of the death penalty
- The Death Penalty Information Center is a rich resource on the death penalty both in the US and internationally
- Information on challenges to lethal injection in various states, including California and North Carolina, is available from the University of California, Berkeley School of Law
- Human Rights Watch monitors executions in the US
- Amnesty International campaigns against the death penalty
- A compendium of death penalty-related links are available from a pro-death-penalty site, the Clark County Prosecuting Attorney

## Introduction

In the United States, lethal injection can be imposed in 37 states and by the federal government and military. The origin of the lethal injection protocol can be traced to legislators in Oklahoma searching for a less expensive and potentially more humane alternative to the electric chair [1]. Both the state medical examiner and a chairman of anesthesiology appear to have been consulted in writing of the statute. The medical examiner has since indicated that no research went into his choice of drugs—thiopental, pancuronium bromide, and potassium chloride—but rather he was guided by his own experience as a patient [2]. His expectation was that the inmate would be adequately anesthetized, and that although each individual drug would be lethal in the dosage specified, the combination would provide redundancy. The anesthesiologist's input relating to thiopental was written into law as “the punishment of death must be inflicted by continuous, intravenous administration of a lethal quantity of an ultra-short-acting barbiturate in combination with a chemical paralytic agent” [3], although in practice Oklahoma uses bolus dosing of all three drugs [4,5]. Texas, the first state to execute a prisoner by lethal injection, and subsequently other jurisdictions, copied Oklahoma's protocol without any additional medical consultation [1].

Although executioners invariably achieve death, the mechanisms of death and the adequacy of anesthesia are unclear. Used independently in sufficiently high doses, thiopental can induce death by respiratory arrest and/or circulatory depression, pancuronium bromide by muscle paralysis and respiratory arrest, and potassium chloride by cardiac arrest. When used together, death might be achieved by a combination of respiratory arrest and cardiac arrest due to one or more of the drugs used. Because thiopental has no analgesic effects (in fact, it can be antianalgesic) [6], and because pancuronium would prevent movement in response to the sensations of suffocation and potassium-induced burning, a continuous surgical plane of anesthesia is necessary to prevent extreme suffering in lethal injection.

Recently we reported that in most US executions, executioners have no anesthesia training, drugs are administered remotely with no monitoring for anesthesia, data are not recorded, and no peer review is done [7]. We suggested that such inherent procedural problems might lead to insufficient anesthesia in executions, an assertion supported by low postmortem blood thiopental levels and eyewitness accounts of problematic executions. Because of a current lack of data and reports of problems with lethal injection for executions, we sought to evaluate the three-drug protocol for its efficacy in producing a rapid death with minimal likelihood of pain and suffering.

## Methods

North Carolina lethal injection protocols were determined from Department of Corrections drug procurement records and testimony of prison personnel participating in the process. Times to death were determined from North Carolina Department of Corrections documents including the Web site [8], official statements, and corroborating news and eyewitness reports. Start times were available for 33 executions, of which 19 could be independently confirmed. The North Carolina warden pronounces death after a flat line is displayed on the electrocardiogram (ECG) monitor for 5 min, thus time to death was calculated from start time to pronouncement of death less 5 min. Dosages were calculated from postmortem body weights taken from Reports of Investigation by the North Carolina Office of the Chief Medical Examiner. Information regarding the California protocol and execution logs and Florida and Virginia executions were obtained through available court documents [9,10,11]. Data are expressed as mean ± standard deviation. One-way ANOVA with Tukey's multiple comparison test was used for statistical analysis.

## Results

### Data from North Carolina Executions

**Figure 1:**
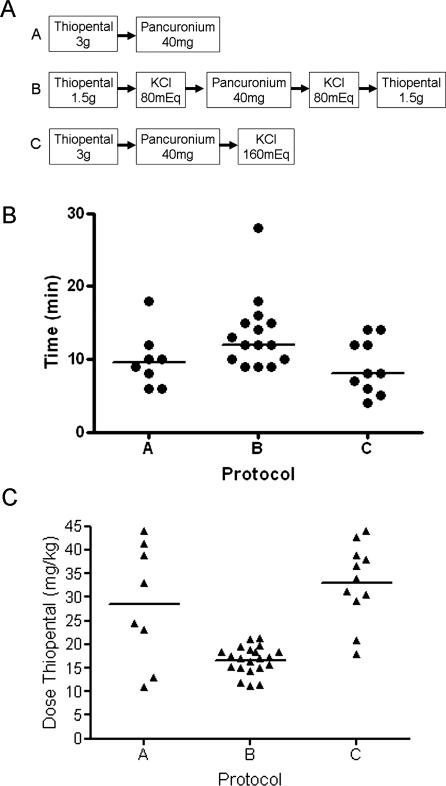
Lethal Injection Executions in North Carolina (A) Schematic depicting quantity and order of drug administration in the three protocols. (B) Time to death by protocol, calculated as the interval from execution start time to declaration of death, minus 5 min (see Methods). (C) Actual dose of thiopental by body weight (not available for all inmates). In Protocol B, 1.5 g of thiopental was given after the pancuronium bromide and potassium chloride, once painful stimuli had been administered and death should have occurred; accordingly, only the first 1.5 g dose is plotted.

Three lethal injection protocols have been used in North Carolina from the first execution in 1984 to the most recent at the time of this writing in August 2006 (Figure 1A). The initial use of serial, intravenous (IV) injections of 3 g of thiopental and 40 mg of pancuronium bromide (referred to here as “Protocol A,” *n* = 8, Figure 1A) was superseded by Protocol B in 1998. Protocol B consisted of serial injections of 1.5 g of thiopental, 80 mEq of potassium chloride, 40 mg of pancuronium bromide, 80 mEq of potassium chloride, and finally 1.5 g of thiopental (*n* = 21) [1,12]. After criticism from expert witnesses [13], in 2004 the injection order was changed to the current protocol of serial injections of 3 g of thiopental, 40 mg of pancuronium bromide, and 160 mEq of potassium chloride (Protocol C, *n* = 11) [14]. Each injection is performed in rapid succession with intermittent saline flushes to avoid drug precipitation. Until the last two executions in 2007, no assessment or monitoring of anesthesia was performed.

According to the North Carolina Department of Corrections, once the ECG monitor displays a flat line for 5 min, the warden declares death and a physician certifies that death has occurred [7, 12]. Execution start times and declaration times were available for 33 of the 42 lethal injections conducted in North Carolina (Figure 1B). Mean times to death were 9.88 ± 3.87 min for Protocol A, 13.47 ± 4.88 min for Protocol B, and 9.00 ± 3.71 min for Protocol C. The mean time to death for Protocol B was significantly longer than for Protocol C (*p* < 0.05, Tukey-Kramer test after one-way ANOVA). No other differences were statistically significant. These data indicate that the five-dose regimen of Protocol B slightly prolonged time to death, but more importantly, they indicate that the addition of potassium chloride did not hasten death overall.

In contrast to clinical use of these same drugs, jurisdictions invariably specify mass quantities for injection rather than dosing by body weight. We sought to determine the actual doses used in executions using postmortem body weights recorded by the Office of the Medical Examiner. North Carolina injects 3 g of thiopental; however, in Protocol B inmates were given half the thiopental at the end, once all painful stimuli were administered and death should have been achieved. Thus we considered only the first 1.5 g for Protocol B. Overall the median thiopental dose was 20.3 mg/kg (range 11.2–44 mg/kg, *n* = 40) (Figure 1C). Virtually all of the lowest doses were under Protocol B, although four very large individuals executed under Protocols A and C received less than the median dose. Eyewitness reports of inmate movement including convulsions and attempts to sit up in four executions [15] did not cluster in the lowest doses, but rather occurred at doses of 17.1, 18.9, 19.6, and 21 mg/kg, all performed under Protocol B. Calculated median doses of pancuronium bromide and potassium chloride were 0.46 mg/kg (range 0.28–0.46 mg/kg) and 1.83 mEq/kg (range 1.11–2.35 mEq/kg), respectively.

### Data from California Executions

**Figure 2:**
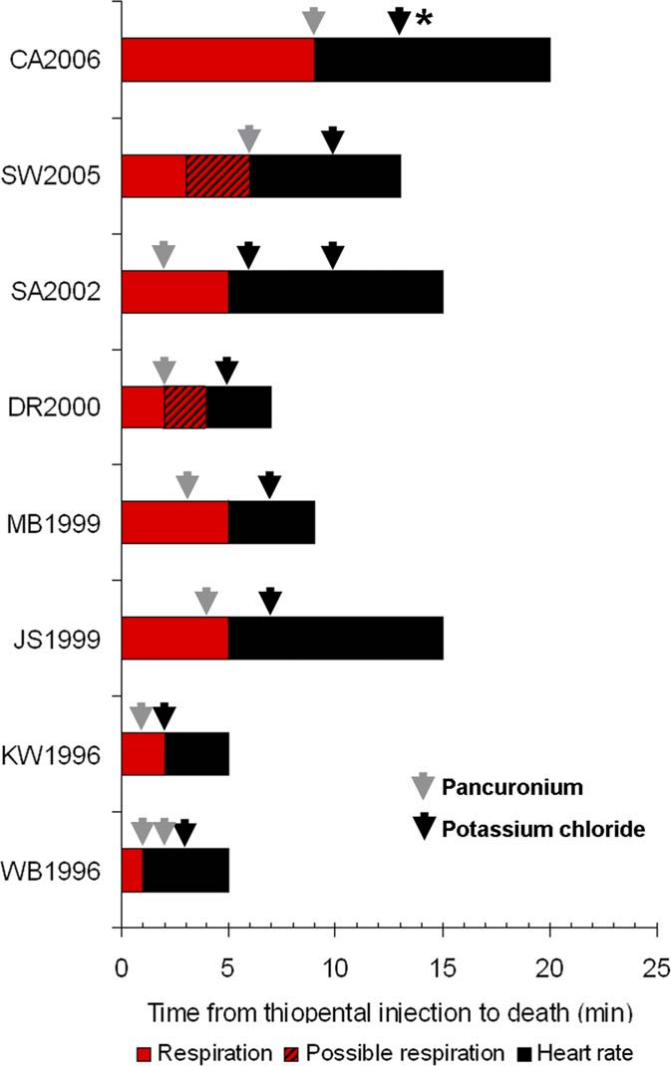
Lethal Injection Executions in California Depicted are duration of respiration and heart rate after initiation of the thiopental injection at time 0. Injection of pancuronium bromide is indicated by the grey arrow, potassium chloride by the black arrow. Note that additional injections of potassium chloride in SA2002 and of pancuronium bromide in WB1996. SW2005 was noted to be breathing 3 min after thiopental, but not at the time of pancuronium bromide injection; the exact time respiration ceased was not recorded. DR2000 was noted to have chest movements two minutes after respiration was noted to have ceased. *A second dose of potassium chloride were administered to CA2006, but not noted on the log. A third, unidentified inmate was also given a second dose of potassium chloride, according to the warden (see text).

Executions in California provided a second insight into the methodologies and outcomes in lethal injections. The public version of the California protocol specifies injection of 5 g of thiopental, 100 mg of pancuronium bromide, and 100 mEq of potassium chloride [9]. California Department of Corrections form 226A, “Lethal Injection—Execution Record,” consists of a table listing “operations,” including injection of each drug, cessation of respiration, flatlining of the cardiac monitor, and pronouncement of death, with columns for time, heart rate, and respiration rate. Such execution records were available for nine of the 11 lethal injections performed in San Quentin California State Prison from 1996 to 2006 [9,10]. One record was incomplete and contradictory and is not reported here. In the remaining eight executions, respiration rate ceased from 1 min (inmate WB1966) to 9 min (CA2006) after the injection of thiopental (Figure 2). Cessation of respiration was noted coincident with (WB1966, SW2005, CA2006) or up to 3 min after (SA2002) injection of pancuronium bromide. Flatlining of the cardiac monitor occurred 2 min (DR2000) to 8 min (JS1999) after the last injection of potassium chloride. The records indicate that a second dose of potassium chloride was used in the execution of SA2002, and the California warden has said that additional doses were used in two other executions, one being CA2006 and the other unknown [16]. Eyewitness reports document “sudden and extreme” convulsive movements 3–4 min into the execution of MB1999 [17] and more than 30 heaving, convulsive movements of the chest and abdomen of SA2002 [18].

## Discussion

Most US executions are beset by procedural problems that could lead to insufficient anesthesia in executions. This hypothesis has been supported by findings of low postmortem blood thiopental levels and eyewitness accounts of problematic executions. Herein we report evidence that the design of the drug scheme itself is flawed*.* Thiopental does not predictably induce respiratory arrest, nor does potassium chloride always induce cardiac arrest. Furthermore, on the basis of execution data and clinical, veterinary, and laboratory animal studies, we posit that the specified quantity of thiopental may not provide surgical anesthesia for the duration of the execution. Thus some inmates may experience the sensations of pancuronium-induced paralysis and respiratory arrest.

In the United States and Europe, techniques of animal euthanasia for clinical, laboratory, and agricultural applications are rigorously evaluated and governed by professional, institutional, and regulatory oversight. In university and laboratory settings, local oversight bodies known as Animal Care and Use Committees typically follow the American Veterinary Medical Association's guidelines on euthanasia, which consider all aspects of euthanasia methods, including drugs, tools, and expertise of personnel in order to minimize pain and distress to the animal. Under those guidelines, lethal injections of companion or laboratory animals are limited to injection by qualified personnel of certain clinically tested, Food and Drug Administration–approved anesthetics or euthanasics, while monitoring for awareness.

In stark contrast to animal euthanasia, lethal injection for judicial execution was designed and implemented with no clinical or basic research whatsoever. To our knowledge, no ethical or oversight groups have ever evaluated the protocols and outcomes in lethal injection. Furthermore, there are no published clinical or experimental data regarding the safety and efficacy of the three-drug lethal injection protocol. Until the unprecedented and controversial use of bispectral index monitoring in the last two North Carolina lethal injections [19], no monitoring for anesthesia was performed. Given this paucity of knowledge and documentation, we sought to evaluate available data in order to determine the efficacy of the three drug protocol.

The designers of lethal injection intended that each of the drugs be fatal independently and that the combination provide redundancy [2]. Moreover, in legal challenges to the death penalty, the leading expert witness testifying on behalf of the states routinely asserts that 3 g of thiopental alone is a lethal dose in almost all cases [14]. The data presented here, however, suggest that thiopental alone might not be lethal. First, extrapolating from clinical use, the lowest dosages used in some jurisdictions would not be expected to kill. Calculated dosages in North Carolina executions using 3 g of thiopental ranged from 10 to 45 mg/kg. Assuming inmates are roughly the same size across jurisdictions, the dose range would be 17–75 mg/kg in California, where 5 g of thiopental is used, and 6.6–30 mg/kg in Virginia and other jurisdictions, which use 2 g. Thus, at the lowest doses, thiopental would be given near the upper range of that recommended for clinical induction of anesthesia (3–6.6 mg/kg)—clearly not a dose designed to be fatal [20]. Second, the calculated doses used across lethal injections are only 0.1–2 times the LD_50_ (dose required to kill 50% of the tested population) of thiopental in dogs (37 mg/kg), rabbits (35 mg/kg), rats (57.8 mg/kg), and mice (91.4 mg/kg) [21, 22]. Third, intravenous delivery of thiopental alone is not recommended by The Netherlands Euthanasics Task Force, which concluded “it is not possible to administer so much of it that a lethal effect is guaranteed” [23], even in their population of profoundly ill patients.

The most compelling evidence that even 5 g of thiopental alone may not be lethal, however, is that some California inmates continued to breathe for up to 9 min after thiopental was injected. This observation directly contradicts testimony of that state's expert witness, who asserted that “this dose of thiopental sodium will cause virtually all persons to stop breathing within a minute of drug administration” and that “virtually every person given 5 grams of thiopental sodium will have stopped breathing prior to the administration of the pancuronium bromide” [24]. The witness has made identical statements regarding 3 g of thiopental [14]. Indeed, the clinical literature is replete with examples of patients experiencing respiratory failure after even low doses of thiopental [25]. Others, however, experience merely transient, nonfatal apnea. Of course, for inmates who did not stop breathing with thiopental alone, it is impossible to know whether the thiopental solution was correctly mixed, whether the entire dose was administered intravenously, or whether the apparent resistance was due to bolus dosing or individual variation. It remains possible, however, that bolus dosing of 5 g of thiopental alone might not be fatal in all persons. Indeed, nonhuman primates given as much as 60 mg/kg (the mass equivalent of 6 g for a 100 kg man) experienced prolonged sleep, but ultimately recovered [26].

If thiopental does not reliably kill the inmates, then perhaps death is effected by potassium chloride. Rapid intravenous or intracardiac administration of 1–2 mmol/kg potassium chloride under general anesthesia is considered acceptable for euthanasia of large animal species; thus the 1.11–2.35 mmol/kg doses given in North Carolina's lethal injections ought to be fatal. If potassium chloride contributes to death through cardiotoxicity, however, cardiac activity ought to cease more quickly when potassium is used than when it is not. Indeed, such is the principle behind the animal euthanasia agent, Beuthanasia-D Special, in which the cardiotoxic effects of phenytoin synergize with the central nervous system–depressive effects of pentobarbital, accelerating death over pentobarbital alone [27]. In contrast, our analysis shows that use of potassium chloride in North Carolina's Protocol C did not hasten death (defined as flatlining of the ECG) over Protocol A, which used thiopental and pancuronium alone. Moreover, in California executions, ECG flatlining was noted from 2 to 9 min after potassium chloride administration. This observation contrasts sharply with reports of accidental bolus IV administration of concentrated potassium chloride solution, in which patients experienced complete cardiopulmonary arrest almost immediately upon injection [28]. The North Carolina and California data together suggest that potassium chloride might not be the lethal agent in lethal injection.

Given that neither thiopental nor potassium chloride can be construed reliably to be the agent of death in lethal injection, death in at least some inmates might have been due to respiratory cessation from the use of pancuronium bromide. The typical use of 0.06–0.1 mg/kg pancuronium bromide under balanced anesthesia produces 100% neuromuscular blockade within 4 min, with approximately 100 min required for 25% recovery [29]. The doses used in North Carolina were some 3–11 times greater than the typical intubation dose, and thus would be expected to produce more rapid paralysis of many hours duration and complete respiratory arrest [30]. Indeed, pancuronium might have been the agent of death even in inmates who ceased breathing coincident with or shortly after injection of pancuronium, rendering permanent the thiopental-induced apnea. In addition, because pancuronium bromide is effective even when delivered subcutaneously or intramuscularly, pancuronium is likely the sole agent of death when IV catheter misplacement or blowout impairs systemic delivery of the other two drugs. In such cases death by suffocation would occur in a paralyzed inmate fully aware of the progressive suffocation and potassium-induced sensation of burning. This was likely the experience of Florida inmate Angel Diaz, whose eyes were open and mouth was moving 24 min into his execution and who was pronounced dead after 34 min. Findings of two 30-cm burns over both antecubital fossae prompted the medical examiner to conclude that the IV lines were misplaced and the drugs were delivered subcutaneously [31].

Executions such as Diaz's, in which additional drugs were required, constitute further evidence that the lethal injection protocols are not adequate to ensure a predictable, painless death. Court documents and news reports indicate that at least Virginia [32], California [10], and Florida [31] have administered additional potassium chloride in multiple executions when the inmate failed to die as expected. If a Virginia execution takes too long and if the inmate fails to die, the protocol indicates that additional pancuronium and potassium chloride should be injected, although there is no provision for additional thiopental [32]. In cases such as Diaz's, additional drugs may have been required due to technical problems with delivery, but it remains possible that in others, the standard drug protocol failed to kill.

Given the uncertainty surrounding the mechanism of death and low postmortem blood thiopental levels in some executed inmates [7], one must ask whether adequate anesthesia is maintained to prevent awareness and suffering. Medical experts on both sides of the lethal injection debate have asserted that 3 g of thiopental properly delivered should reliably result in either death or a long, deep surgical plane of anesthesia [13,14]. In support of this contention, continuous or intermittent thiopental administration was formerly used for surgical procedures lasting many hours. In one study, 3.3–3.9 g given to patients over 25–50 min resulted in sleep for 4–5.5 h [33]. Depth and duration of thiopental anesthesia depends greatly upon dose and rate of administration, however, and bolus dosing results in significantly different pharmacokinetics and duration of efficacy than administration of the same quantity of drug at a lower rate [22].

In the modern practice of anesthesia, thiopental is used solely to induce a few moments of anesthesia prior to administering additional agents. Anesthesiologists are taught to administer a small test dose while assessing patient response and the need for additional doses [20]. Such stepwise administration and evaluation has been the practice from the first reports of thiopental usage in 1934, due to the known potential for barbiturate-induced respiratory arrest [34]. It was early recognized that age, body composition, health status, anxiety, premedication, and history of substance abuse clearly influence response to thiopental, with some individuals showing marked resistance to standard doses [35] and others fatal sensitivity [25]. Thus the historical and modern clinical use of thiopental results from its cautious application to prevent respiratory arrest both in the typical patient and the abnormally susceptible. In consequence, there is almost no information about duration of anesthesia following large bolus doses of thiopental in unpremedicated patients, and there are few living anesthesiologists with clinical experience relevant to lethal injection protocols.

Unlike in clinical medicine, however, bolus injection of thiopental is regularly practiced in laboratory animals and veterinary medicine. Standard texts specify from 6 to 50 mg/kg thiopental, depending on the species, for 5–10 min of anesthesia [36], including 18–22 mg/kg for 10–15 min of anesthesia in dogs, pigs, sheep, and swine [37]. Such dosages are conservative guidelines based on average responses of animals in experimental trials (Table 1), with the assumption that respiration and depth of anesthesia will be assessed in individual animals prior to onset of the procedure. (In addition, thiopental is not recommended for painful procedures in animals.) Withholding or administering additional dosages would compensate for individual variation in response.

**Table 1:**
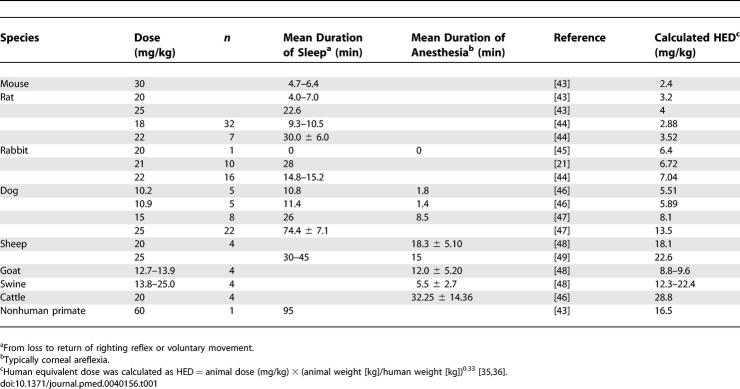
Reported Duration of Sleep or Anesthesia after Bolus IV Injections of Thiopental in Experimental Animals

Although species differences complicate pharmacological comparisons from animals to humans, animal studies are the basis for virtually all human drug trials. According to FDA guidelines, toxicity endpoints for drugs administered systemically to animals are typically assumed to scale well across species when doses are normalized to body surface area (i.e., mg/m^2^) [38]. Calculating the human equivalent dose (HED) as recommended by the FDA [39] gives a more conservative estimate of thiopental equivalencies across species than does using simple mg/kg comparisons (Table 1). Swine in particular are regarded as an excellent model of human cardiopulmonary and cerebrovascular physiology, with comparable size, body composition, and brain perfusion rates [40]. Comparing the HED for thiopental anesthesia in swine to lethal injection dosages, we conclude that at least some inmates at the lower end of the thiopental dose range might have experienced fleeting or no surgical anesthesia, while others at the higher end of the range might have received doses predicted to induce more prolonged anesthesia (Table 1). Such a prediction is impossible to evaluate, however, because any evidence of suffering would be masked by the effects of pancuronium.

Our study is necessarily limited in scope and interpretations. Given the secrecy surrounding lethal injections, we were able to analyze only a small fraction of the 891 lethal injections in the US to date. Indeed, the majority of executions actually take place in states such as Texas and Virginia, where the protocols and procedural problems are likely similar to the ones described, but where the states are unwilling to provide information [7]. Not only are available data limited, however, medical literature addressing the effects of these drugs at high doses and in combination is nonexistent, emphasizing the failure of lethal injection practitioners to design and evaluate rigorously a process that ensures reliable, painless death, even in animals. In consequence, the adequacy of anesthesia and mechanism of death in the current lethal injection protocol remains conjecture.

Despite such limitations, our analysis of data from more forthcoming states along with reports of problematic executions and judicial findings [41] together indicate that the protocol of lethal injection for execution is deeply flawed. Technical difficulties are clearly responsible for some mishandled executions, such as Diaz's. Better training of execution personnel and altering delivery conditions may not “fix” the problem [41, 42], however, because the drug regimen itself is potentially inadequate. Our analysis indicates that as used, thiopental might be insufficient both to maintain a surgical plane of anesthesia and to predictably induce death. Consequently, elimination of pancuronium or both pancuronium and potassium, as has been suggested in California [41], could result in situations in which inmates ultimately awaken.

With the growing recognition of flaws in the lethal injection protocol, 11 states have now suspended the death penalty, with nine of those seeking resolution of issues surrounding the process [42]. In California and Florida, commissions of experts have been charged with evaluating and refining lethal injection protocols. As deliberations begin, we suggest that the secrecy surrounding protocol design and implementation should be broken. The available data or lack of data should be made public and deliberations should be open and transparent.

## Supporting Information

Alternative Language Abstract S1 Translation into Spanish by Francisco López-Muñoz (24 KB DOC)

## Abbreviations

ECG: electrocardiogram
HED: human equivalent dose
IV: intravenous
