# Supplementary material for: Lethal Injection for Execution: Chemical Asphyxiation?
Source: PLoS Med. 2007 Apr 24;4(4):e156. doi: 10.1371/journal.pmed.0040156 (PMC1876417; doi:10.1371/journal.pmed.0040156)
Supplement: Alternative Language Abstract S1 — (24 KB DOC) [file pmed.0040156.sd001.doc]

**Ejecución por inyección letal: ¿Asfixia química?**

**Resumen**

**Antecedentes**: La ejecución por inyección letal fue concebida como una alternativa más humana a las ejecuciones por electrocución o por aplicación de cianuro. Los protocolos actuales están basados en una supervisión improvisada por parte de un examinador médico y un anestesista en Oklahoma y son realizados a discreción de los funcionarios de prisiones. Cada uno de los fármacos integrados en la inyección (el tiopental, un barbitúrico de acción ultracorta, el bloqueante neuromuscular bromuro de pancuronio, y el cloruro potásico, un agente electrolítico) poseen potencialidad letal por sí mismos, mientras que con la combinación se pretende inducir anestesia primero y luego la muerte por parada cardiorrespiratoria.

**Métodos y Resultados**: Hemos analizado los datos procedentes de ejecuciones en Carolina del Norte y California, así como los datos de experimentación animal, a nivel de clínica veterinaria. Los resultados de las ejecuciones, junto al variable efecto entre especies de distintas dosis de tiopental, sugieren que, en la práctica habitual de la inyección letal, el tiopental podría no ejercer un efecto fatal y ser insuficiente para inducir una anestesia quirúrgica duradera durante el periodo de la ejecución. Es más, existen evidencias de ejecuciones en Carolina del Norte, California y Virginia que indican que el cloruro potásico contenido en las inyecciones letales no induce realmente una parada cardiaca.

**Conclusiones**: Los protocolos actuales sobre inyección letal no causan un efecto mortal mediante los mecanismos pretendidos, lo que indica un fallo de su diseño e implementación. Si los efectos del tiopental y del cloruro potásico son insuficientes, podríamos encontrarnos frente al fallecimiento de los reclusos por asfixia inducida por pancuronio. Asimismo, desde el punto de vista pragmático, el hecho de que la inyección letal sea un método de ejecución indoloro y pacífico es cuestionable.
